# Impact of the SARS-CoV-2 (COVID19) pandemic on the morbidity and mortality of high risk patients undergoing surgery: a non-inferiority retrospective observational study

**DOI:** 10.1186/s12871-021-01495-3

**Published:** 2021-11-26

**Authors:** Marta Caballero-Milán, Maria J. Colomina, Leo A. Marin-Carcey, Laura Viguera-Fernandez, Roser Bayona-Domenge, Sara Garcia-Ballester, Albert López-Farre, Leticia Ruiz-Buera, Maite Sanz-Iturbe, David Álvarez-Villegas, Ely C. Jenssen-Paz, Guillermo Puig-Sanz, Aaron Arcos-Terrones, Carmen Belmonte-Cuenca, Elia Perelada-Alonso, Francho Blasco-Blasco, Antoni Sabaté

**Affiliations:** 1 Department of Anaesthesiology and Critical Care, Bellvitge University Hospital, University of Barcelona, Hospitalet de Llobregat, carrer Feixa Llarga s/n, 08907 Barcelona, Spain; 2 Health Group, Neuroscience Program, Perioperative Physiopathology and Pain, Institut d’Investigació Biomèdica de Bellvitge – IDIBELL, Hospitalet de Llobregat, Barcelona, Spain; 3 Department of Clinical Sciences, School of Medicine, Universitat de Barcelona – UB, L’hospitalet de Llobregat (Barcelina), Barcelona, Spain

**Keywords:** Elective surgery, Emergent surgery, SARS-CoV-2 (COVID19) pandemic, Clavien-Dindo complications, Mortality

## Abstract

**Background:**

During the COVID-19 crisis it was necessary to generate a specific care network and reconvert operating rooms to attend emergency and high-acuity patients undergoing complex surgery. The aim of this study is to classify postoperative complications and mortality and to assess the impact that the COVID-19 pandemic may have had on the results.

**Methods:**

this is a non-inferiority retrospective observational study. Two different groups of surgical patients were created: Pre-pandemic COVID and Pandemic COVID. Severity of illness was rated according to the Diagnosis-related Groups (DRG) score. Comparisons were made between groups and between DRG severity score-matched samples. Non-inferiority was set at up to 10 % difference for grade III to V complications according to the Clavien-Dindo classification, and up to 2 % difference in mortality.

**Results:**

A total of 1649 patients in the PreCOVID group and 763 patients in the COVID group were analysed; 371 patients were matched for DRG severity score 3-4 (236 preCOVID and 135 COVID). No differences were found in relation to re-operation (22.5 % vs. 21.5 %) or late admission to critical care unit (5.1 % vs. 4.5 %). Clavien grade III to V complications occurred in 107 patients (45.3 %) in the PreCOVID group and in 56 patients (41.5 %) in the COVID group, and mortality was 12.7 % and 12.6 %, respectively. During the pandemic, 3 % of patients tested positive for Covid-19 on PCR: 12 patients undergoing elective surgery and 11 emergency surgery; there were 5 deaths, 3 of which were due to respiratory failure following Covid-19-induced pneumonia.

**Conclusions:**

Although this study has some limitations, it has shown the non-inferiority of surgical outcomes during the COVID pandemic, and indicates that resuming elective surgery is safe.

**Trial registration:**

Clinicaltrials.gov identifier: NCT04780594.

**Supplementary Information:**

The online version contains supplementary material available at 10.1186/s12871-021-01495-3.

## Introduction

In December 2019, the disease produced by a new SARS-CoV-2coronavirus, named COVID-19 (Coronavirus Disease 2019), was detected in Wuhan (Hubei Province, China). Since then, the disease has spread rapidly worldwide and was declared a pandemic by the WHO in March 2020.

Spain is among the countries with the highest rate of infection. The latest reports at the end of the first wave of the pandemic (24 May 2020) refer to a total of 235,290 confirmed cases, and a high percentage of hospitalisations for both conventional and critical care.

During the peak of the COVID-19 health emergency, from 11 to 2020 to 15 May 2020, the ratio of patients admitted to hospital was nearly 200 per million population; consequently the number of beds dedicated to COVID-19 infected patients increased. In various wards (Pneumology, Internal Medicine and Infectious Medicine), the highest number of COVID-19 patients admitted in one day during the pandemic was 396, and the number of critical care beds reserved for COVID-19 patients increased to 108.

Low and intermediate elective surgeries were postponed during the pandemic, but high-acuity cases were not, so it was necessary to create a specific care network to attend to non-COVID-19 patients requiring emergency care, critical care, and major/complex surgery, and reconvert operating rooms into critical care beds. This re-organization allowed hospitals to continue with emergency surgical procedures and major oncological and cardiovascular surgery procedures in patients at risk of decompensation. In this context, triage may benefit time-sensitive outcomes in elective surgery.

Various authors have reported that during the pandemic, mortality among COVID-19-infected patients was higher than the rate associated with the same surgical procedures in the pre-pandemic period [1, 2]. Our working hypothesis, however, is that outcomes in our hospital have not differed in patients operated during vs. before the pandemic. The aim of this study is to evaluate the complications and mortality in patients operated during the COVID crisis. We sought to demonstrate that the pandemic had no negative impact on surgical outcomes in terms of morbidity and mortality when protective protocols for both patients and health care personal were implemented.

We performed this non-inferiority study in healthcare outcomes by comparing 2 periods, pre-pandemic and pandemic. This comparison is also intended to determine quality of healthcare in the operating room. The analysis of our data will help us determine which measures are essential in future COVID-19 crises.

## Methods

### Ethics

Ethical approval for this study (N° PR227/20) was provided by the Ethical Committee of Bellvitge University Hospital, Hospitalet de Llobregat, Barcelona, Spain (Chairperson Prof E. Sospedra) on 25 June 2020. Patients were asked to sign an informed consent form to use their data at the time of hospital admission.

### Study design

This is a non-inferiority retrospective observational study. Once the first wave had passed, we decided to analyse the impact that the COVID-19 pandemic had had on our surgical program and on postoperative outcomes. To this end, surgical activity during the pandemic period was compared with a similar period prior to the pandemic.

We also intend to highlight the role of the anaesthesiologists in planning the surgical schedule, selecting patients, and adapting critical care bed capacity to meet surgical needs during the pandemic.

### Inclusion criteria

Using automatically generated data from the minimum data set for surgical procedures that includes all surgical patients (elective and emergent cases), 2 different groups of surgical patients were created:

- Pre-pandemic group (PreCOVID): All patients who underwent surgery from 13 January to 29 February 2020, far enough from removed from the pandemic to avoid including COVID-19-infected patients.
- Pandemic group (COVID): All patients who underwent surgery from 11 to 2020 to 15 May 2020, during the first wave of the pandemic.

### Exclusion criteria

1. Minor to intermediate surgery performed in the pre-pandemic period that involves discharge from the hospital on the same day or the following day.
2. Procedures related to medical treatment or complications of COVID-19 patients, such as chest tubes, extracorporeal oxygenation or tracheostomy.
3. Surgery scheduled during the COVID period in patients whose pre-operative RT-PCR test was positive. In these cases, surgery was postponed until the patients were negative, but they were not eligible for inclusion in the study even if they eventually underwent surgery.

### Setting and management in the COVID Period

Several organisational changes were made to the surgical pathway: the sICU and trauma-ICU were moved to the operating room (OR) area for non-COVID patients, and were reduced from 24 critical beds in the pre-pandemic period to 11 critical beds in the COVID period. Other measures were preoperative online symptomatic screening for COVID in scheduled surgery patients and RT-PCR screening for emergency patients. All spaces in the OR area were clearly differentiated: clean COVID OR, COVID OR, and clean ICU; different entry and exit routes were used for the OR Area and sICU to ensure the operating room area was safe for surgery and postoperative and critical management. A specific COVID OR was used for emergency surgery patients with a positive RT-PCR; after the procedure patients were admitted to a COVID-ICU. An airway management protocol was implemented, and the use of personal protective equipment was mandatory for all staff involved in the OR and sICU OR.

Surgery departments triaged elective surgery patients for high acuity cases, and these were discussed at the weekly planning meeting if Covid-19 screening was negative. The anaesthesiologists coordinated the surgical schedule, and adjusted the critical bed capacity to surgical needs. After surgery, all patients were screening for Covid-19 infection by RT-PCR.

### Data collection

Data on patient demographics, clinical history, surgical team and surgical procedure, primary and secondary diagnoses, and primary and secondary surgical procedures were collected automatically. A case-mix grouping system based on diagnosis related groups using ICD-10-AM International Classification of Diseases nomenclature was created. The Severity of Illness Index assigns to each patient an overall severity score (from 1 to 4) and mortality risk score (from 1 to 4). [3].

The primary end point was the percentage of patients with grade III to V complications according to the Clavien-Dindo classification.

The relevant variables analysed for all patients included in both periods were the following:

- Age.
- Sex.
- Severity according to the DRG scale.
- Risk according to the DRG scale.
- Day of admission to the hospital.
- Day of surgery.
- Diagnosis of the pathology.
- Type of surgical intervention by specialty and timing (scheduled or emergency).
- Per-protocol admission to the sICU (scheduled surgery).
- Late, non-scheduled admission to sICU.
- Length of hospital stay.
- Positive RT-PCR screening for Infection by COVID-19.
- Surgical complications according to the Clavien –Dindo classification.
- In-hospital mortality.
- Discharge destination (home, death, or nursing home).

Data were collected from the administrative database in November 2020, so all patients were followed up until they were discharged from the hospital. Additionally, in patients with high risk or DRG severity score (3 and 4) and emergent procedures the full clinical history was analysed and the cause of death was determined by consensus between the main study investigators (MC, MJC, and AS).

Patients in whom preoperative or postoperative COVID-19 testing resulted positive (diagnosis confirmed by RT-PCR) were also analysed on the basis of their full clinical history. A nosocomial infection was considered if patients manifested COVID-19 symptoms from the third day of admission until discharge.

### Sample size

During the Covid period, a total of 763 patients were operated. In order to determine the size of the sample, we anticipated a 15 % Clavien III-V complication rates among patients during this period and an inclusion ratio of 2:1 with a power of 90 % and a significant alfa level of 5 %, resulting in 1527 patients to be analysed from the PreCovid period.

### Statistical analysis

Comparisons were made between the PreCOVID group and the COVID group. In order to avoid bias, a selected matched sample on the basis of the DRG severity score was created in both groups and compared. The results were analysed after selecting matched patients.

A descriptive analysis was performed using the usual statistical tests: Chi-square tests to compare the categorical variables, and parametric or non-parametric tests for continuous variables (depending on their normality). For all variables, bilateral tests with a significance level of 5 % were used. Criteria for Non-inferiority were: up to 10 % difference for grade III to V complications according to the Clavien-Dindo classification, and up to 2 % difference for mortality.

## Results

### Participants

During the COVID period, 21 patients were excluded from the analysis because the procedure performed involved medical patients with COVID-19 infection (pleural procedure, tracheostomy and extracorporeal oxygenation). In total, 2412 patients were analysed during both study periods. In the PreCOVID period, 1649 patients were operated (PreCOVID group) and 763 patients in the COVID group. Four subgroups were created on the basis of the type of surgery:

1. Major scheduled surgery performed during the preCOVID-19 period.
2. Emergency surgery performed during the preCOVID-19 period.
3. Major elective surgery performed during the COVID 19 period.
4. Emergency surgery performed during the COVID-19 period.

Comparisons between the PreCOVID group and COVID group are shown in Table 1. Most patients in the COVID group were women with greater severity and higher DRG risk scores; more urologic procedures were performed and fewer general and digestive surgeries. Fewer patients in the COVID group were admitted per protocol to the sICU, but more required late admission to the sICU and had higher Clavien-Dindo scores; however, hospital length of stay, the percentage of patients discharged home and non-survivors did not differ (Table 1).

**Table 1:** Patient demographics and outcomes in both study periods (preCOVID and COVID groups)

PERIOD	PRECOVID (n= 1649)	PRECOVID ELECTIVE (n= 1490)	PRECOVID EMERGENT (n=159)	COVID (n=763)	COVID ELECTIVE (n=585)	COVID EMERGENT(n=178)	PRECOVID vs. COVID (p value)
Age, years (IC 95 %)	62.2 (61.3 – 62.7)	62 (61.3 - 62.8)	63.7 (60.7 – 66.7)	66.1 (64.6 – 67.7)	63.1 (61.8 – 64.4)	64.6 (61.9 – 67.4)	0.065
Male sex, n (%)	899 (54.5)	807 (54.2)	92 (57.9)	379 (49.7)	281 (48)	98 (55.1)	0.028
SPECIALTY							0.018
Digestive and Gynaecological, n (%)	461 (28)	391 (26.2)	70 (44)	177 (23.2)	125 (21.4)	52 (29.2)	
Orthopaedics and Trauma, n (%)	294 (17.8)	264 (17.7)	30 (18.9)	140 (18.3)	85 (14.5)	55 (30.9)	
Plastic and Neck, n (%)	288 (17.5)	276 (18,5)	12 (7.6)	148(19.4)	130 (22.2)	18 (10.1)	
Cardiovascular and thoracic, n (%)	291 (17.6)	271 (18.2)	20 (12.6)	138 (18.1)	112 (19.2)	26 (14.6)	
Neurosurgery, n (%)	160 (9.7)	138 (9,3)	22 (13.8)	69 (9.1)	50 (8.5)	19 (10.7)	
Urology, n (%)	155 (9.4)	150 (10.1)	5 (3.1)	91 (11.9)	83 (14.2)	8 (4.5)	
DRG SEVERITY							
1-2 (%)	1413 (85.7)	1318 (88.5)	95 (59.7)	650 (82.3)	500 (85.5)	128 (71.9)	0.123
3-4 (%)	236 (14.3)	172 (11.5)	64 (40.3)	135 (17.7)	85 (14.5)	50 (28.1)	0.034
DRG RISK							
1-2, n (%)	1485 (90.1)	1378 (92.5)	107 (67.3)	650 (85.2)	524 (89.6)	126 (70.8)	0.000
3-4, n (%)	164 (9.9)	113 (7.6)	58 (36.5)	113 (14.8)	61 (10.4)	52 (29.2)	0.000
REINTERVENTION, n (%)	138 (8.4)	112 (7.5)	26 (16.4)	55 (7.2)	38 (6.5)	17 (9.6)	0.375
sICU ADMISSION							
Per protocol, n (%)	470 (28.5)	430 (28.9)	40 (25.1)	130 (17)	102 (17.4)	28 (15.7)	0.000
Late admission, n (%)	20 (1.2)	11 (0.7)	9 (5.6)	18 (2.4)	15 (2.6)	3 (1.7)	
CLAVIEN DINDO							
I, n (%)	1445 (87.6)	1370(91.8)	75 (44.8)	580 (76)	503 ( 85.9)	77 (43.3)	
II, n (%)	84 (5.1)	50 (3.4)	34 (20.6)	108 (14.2)	42 (7.2)	66 (37.1)	
IIIa, n (%)	9 (0.5)	4 (0.3)	5 (3)	6 (0.8)	2 (0.3)	4 (2.2)	
IIIb, n (%)	31 (1.9)	20 (1.3)	11 (6.7)	23 (3)	15 (2.6)	8 (4.5)	
IVa, n (%)	25 (1.5)	13 (0.9)	12 (7.3)	16 (2.1)	10 (1.7)	6 (3.4)	
IVb, n (%)	24 (1.5)	15 (1)	9 (5.5)	13 (1.7)	8 (1.4)	5 (2.8)	
V, n (%)	31 (1.9)	18 (1.3)	13 (12.1)	17 (2.2)	5 (0.9)	12 (6.7)	
III-V	120 (7.3)	70 (4.7)	50 (31.4)	75 (9.8)	40 (6.8)	35 (19.7)	0.037
Length of stay (days)	10 (9-11)	9.1 (8-10.1)	18.7 (14.7-22.7)	9.8 (8.3-11.3)	7.6 (6.5-8.7)	17 (11.5-22.5)	0.774
DESTINATION AT DISCHARGE							
Home (%)	63.1	70	58.5	70.9	73.1	63.5	
Death (%)	1.9	1.2	8.2	2.2	0.9	6.7	0.638
Nursing home (%)	353	28.8	33.3	26.9	26	29.8	

Both periods (PRECOVID group and COVID group) are subdivided into elective and emergency surgery. DRG: Diagnosis Related Groups. Statistical analysis: Chi-square tests to compare the categorical variables and the subtraction of variables using parametric or non-parametric tests for continuous variables

In both periods, 48 patients (2 %) died in both groups (additional information in supplementary Tables 1, access on the website), and severe complications (Clavien grade III to V) occurred in 195 patients (8 %). In a multivariate analysis, deaths correlated with age (odds ratio 0.95, 95 % CI 0.924-0.988) and DRG severity score (odds ratio 0.177, 95 % IC 0.08-0.38), but not with the period when the surgical procedure was performed (odds ratio 1.21 95 % IC 0.56-2.6).

### Scheduled surgery (groups 1-3, Table 1)

A total of 1490 patients underwent major elective procedures during the pre-COVID period, and 585 patients in the COVID period. The COVID group was similar in age, were mostly women, with greater severity and higher DRG risk scores, and fewer were admitted per protocol to the sICU. There were no differences in mortality and length of hospital stay. The percentage of patients discharged home was similar (Table 1).

### Emergency surgery (groups 2-4, Table 1)

A total of 159 patients underwent emergency surgery during the pre-pandemic period, and 178 patients in the pandemic period. The DRG risk of mortality was higher in the COVID group. Fewer patients in the COVID group were admitted to the sICU. No differences were found in mortality, discharge home, or length of stay.

### Matched subgroups according to the DRG severity score (Table 2)

**Table 2:** Patient demographics and outcomes between matched DRG severity score 3-4

PERIOD	PRECOVID GROUP (n= 236)	COVID GROUP (n=135)	PRECOVID VS COVID (p value)
Age (years)	66 (64-68)	66 (63.5-69)	0.914
Sex, male (%)	165 (69.9)	75 (55,6)	0.007
SURGERY BY SPECIALTY			0.418
Digestive and Gynaecological, n (%)	53 (22.5)	32 (23.7)	
Orthopaedics and Trauma, n (%)	34 (14.4)	27 (20)	
Plastic and Neck, n (%)	23 (9.7)	18 (13.3)	
Cardiovascular and thoracic, n (%)	79 (33.5)	35 (26)	
Neurosurgery, n (%)	29 (12.3)	15 (11.1)	
Urology, n (%)	18 (7.6)	8 (5.9)	
REINTERVENTION (%)	53 (22.5)	29 (21.5)	0-897
sICU			
Per protocol, n (%)	152 (64.4)	60 (44.4)	0.000
Late admission, n (%)	12 (5.1)	6 (4.4)	
Clavien Dindo			
I, n (%)	67 (28.4)	33 (24.4)	
II, n (%)	62 (26.3)	45 (33.3)	
IIIa, n (%)	8 (3.4)	3 (2.2)	
IIIb, n (%)	26 (11)	15 (11.1)	
Iva, n (%)	21 (8.9)	11 (8.2)	
IVb, n (%)	22 (9.3)	11 (8.2)	
V, n (%)	30 (12.7)	17 (12.6)	
III-V	107 (45.3)	56 (41.5)	0.515
Length of stay (days)	32.7	26.5	0.774
DESTINATION AT DISCHARGE			
Home (%)	45.3	48	
Dead (%)	12.7	12.6	1
Others (%)	42	39.4	

Only the patients with a DRG severity score 3-4 are shown. These are considered the patients with the highest risk of complications. DRG: Diagnosis Related Groups. Chi-square tests to compare the categorical variables, and the subtraction of variables using parametric or non-parametric tests for the continuous variables

In a subgroup of patients matched by DRG severity score 3 and 4, (group 5 and 6), 236 patients were considered DRG severity 3-4 in the PreCOVID group and 135 patients in the COVID group. There were no differences between matched groups in terms of age, emergency procedure, and surgical speciality; however, more men were included in the matched pre-pandemic subgroup, and per protocol admission to the sICU was higher in this subgroup. No differences were found in relation to re-operation (22.5 % vs. 21.5 %), late admission to sICU (5.1 % vs. 4.5 %), or length of hospital stay. Severe complications (Clavien grade III to V) occurred in 107 patients (45.3 %%) in the Pre-COVID group and in 56 patients (41.5 %) in the COVID group, and mortality was 12.7 % and 12.6 %, respectively.

### COVID-19

COVID-19 infection during both periods occurred in 25 patients (Table 3). Two occurred in the pre-pandemic period as a post-operative complication; this was classed as a nosocomial infection.

**Table 3:** COVID patients in preCOVID and COVID groups

Age (years)	Sex	Period	Diagnose	Procedure (*)	Surgery by Specialty	DRG s	COVID Nosocomial infection?	COVID Symptoms?	BRCSS	Need for ICU?	Length of stay (days)	Died?	Cause of death
79,5	0	2	Trochanteric fracture of the femur	Internal fracture fixation	Orthopaedics & Trauma	3	No	Yes	1	No	19.76	No	
90.1	1	2	Right femur neck fracture	Internal fracture reduction	Orthopaedics & Trauma	2	No	Yes	1	No	13.87	No	
69.0	1	2	Non-traumatic bowel perforation	Exploratory laparotomy	Digestive	2	No	Yes	3	Yes	23.71	No	
80.8	1	2	Femur fracture	Partial hip replacement	Orthopaedics & Trauma	1	no	No		No	11.66	No	
90.9	1	2	Coronavirus infection	Internal femur fracture reduction	Orthopaedics & Trauma	3	no	Yes	1	No	19.86	No	
92.1	1	2	Right femur fracture	Internal fracture reduction	Orthopaedics & Trauma	2	No	Yes	1	No	14.50	no	
73.6	0	2	Incisional hernia. with obstruction	Other abdominal surgery	Digestive	4	No	Yes	3	Yes	17.41	Yes	Respiratory failure
70.8	0	2	Acute pancreatitis	Excision or destruction of pancreatic tissue	Digestive	4	Yes	Yes	3	Yes	69.30	Yes	Multiorgan failure
79.3	0	2	Abdominal aortic aneurysm	Intravascular implantation of aorta grafts	Cardiovascular	4	Yes	Yes	3	Yes	15.43	Yes	Respiratory failure
74.7	1	2	Other specific explorations	Left hemicolectomy	Digestive	2	Yes	No		No	17.47	No	
78.4	0	2	Malignant neoplasia of the colon	Right hemicolectomy	Digestive	1	Yes	No		No	8.41	No	
86.5	1	2	Right trochanteric fracture of the femur	Internal fracture reduction	Orthopaedics & Trauma	3	No	No		No	14.54	No	
91.3	1	2	Right femur neck fracture	Internal fracture reduction	Orthopaedics & Trauma	1	No	No		No	5.93	No	
86.6	1	2	Left trochanteric fracture of the femur	Internal fracture reduction	Orthopaedics & Trauma	1	No	No		No	2.89	No	
38.6	1	2	Cerebral aneurysm. without rupture	Other aneurysm repair	Neurosurgery	4	Yes	Yes	3	Yes	112.20	No	
71.6	0	2	Left femur neck fracture	Partial hip replacement	Orthopaedics & Trauma	2	No	No		No	6.64	No	
66.3	0	2	Assistance for other specific explorations	Craniotomy	Neurosurgery	1	Yes	Yes	1	No	8.23	no	
52.8	0	2	Gastrointestinal haemorrhage	Exploratory laparotomy	Digestive	4	Yes	Yes	2	No	60.54	No	
46.4	1	2	Ulcerative colitis	Laparoscopic intra-abdominal colectomy	Digestive	2	Yes	No		No	35.05	No	
52.5	1	2	Malignant breast neoplasia	Bilateral mastectomy	Plastic & Neck	2	Yes	No		No	3.25	No	
36.2	1	2	Vesicovaginal fistula	Permanent colostomy	Orthopaedics & Trauma	2	Yes	No		No	44.00	No	
80.0	1	2	Other circulatory disorders	Below knee amputation	Cardiovascular	1	No	No		No	4.5	No	
72.2	0	1	Right frontoparietal haemorrhagic stroke	Decompressive craniectomy	Neurosurgery	4	Yes	No		Yes	48.00	Yes	Ischemia and bleeding of vascular malformation
85.6	1	1	Periprosthetic knee fracture	Internal fracture reduction	Orthopaedics & Trauma	4	Yes	Yes	4	No	51.79	Yes	Respiratory failure
53.4	0	1	Severe polytrauma	Pelvic osteotaxis	Orthopaedics & Trauma	4	Yes	Yes	2	No	172.00	No	

(*) procedures related to medical treatment or complications of COVID19 are excluded. Sex (0: male, 1:female), Period (1: PRECOVID elective, 2: PRECOVID emergent, 3: COVID elective; 4: COVID emergent), DRG s: Diagnosis Related Groups Severity, BRCSS : Brescia Respiratory COVID19 severity scale

In the pandemic period, 12 patients undergoing elective surgery tested positive for Covid-19 on RT-PCR in the postoperative period; all survived. Eleven patients undergoing emergency surgery tested positive on RT-PCR before surgery; 3 died due to Covid-19-induced pneumonia leading to respiratory failure; 2 other deaths in in this group patients with positive RT-PCR were not related to Covid-19 pneumonia. Overall, 23 patients (3 %) in the COVID group tested positive on RT-PCR.

## Discussion

Overall, patients in the COVID period presented greater severity and higher DRG risk scores, and emergency surgery was more frequent. They had more Clavien III-V complications, but this difference did not reach the significance required to show inferiority. There were no differences in hospital length of stay in patients discharged home and in-hospital deaths with respect to the Pre-pandemic period. Death or disability (Clavien-Dindo IV and V) occurred in 4.8 % in the COVID group vs. 6.1 % in the PreCOVID group; this difference was not significant. This percentage in the COVID group is similar to that reported in other studies [4]. However, when comparing GRD severity-matched groups with similar ages and types of surgery (emergency and general surgery), no differences were found in terms of re-operation, Clavien-Dindo complications, length of hospital stay and mortality. These results show the non-inferiority outcome in the COVID population, despite the limitations imposed by the reorganisation of the surgical pathway, particularly the shortage of sICU beds, during the Covid-19 pandemic.

The reduction of surgical critical care beds during the pandemic period clearly limited the number of major elective surgery procedures performed. However, late sICU admission in high severity matched samples was similar in both periods. In non-epidemic conditions, around 40 % of critical beds are used for trauma and surgical patients [5], but only 13 % are used for elective surgery and around 6 % for patients receiving mechanical ventilation following elective surgical procedures [5]. Based on these figures and our experience, even if the number of critical beds were reduced, rationalising the use of the sICU should minimise the need to suspend elective surgery, and should not be a limitation for scheduling more surgical patients in future Covid-19 waves.

In our cohort, age was associated with mortality in a multivariate analysis, a finding similar to other series. Advanced age predicts a 2- to 4-fold increase in morbidity and mortality [6]. Furthermore, age and pre-existing disease severity are factors for 1-year mortality after an acute episode [7]. In our cohort, DRG severity was associated with mortality. The DRG severity score is an indirect but incomplete measure of frailty, which is defined as “a multidimensional syndrome characterized by decreased reserves that leaves an individual vulnerable to adverse outcomes due to decreased tolerance of stressors (physical, physiologic, or psychosocial)” [8]. The prevalence of frailty is higher in emergency vs. elective procedures [9]. Frailty is consistently associated with an increase in the risk of major morbidity, mortality, and re-admissions [10], and increases the odds of non-home discharge among older patients [11].

The re-operation rate was high in both subgroups of patients matched by DRG severity score 3 and 4. This had a major influence on severe complications (Clavien grade III to V) and mortality, which was 12.7 % and 12.6 % respectively. Efforts should be made to improve surgical outcomes, even in patients with severe medical comorbidity. These results are similar to those of the Collaborative study [2], in which age and emergency surgery were significant predictors of mortality.

In the COVID group, 12 asymptomatic patients tested positive on RT-PCR during hospitalization, which represents 2 % of elective surgeries. These patients were not detected in the preoperative clinical screening for Covid-19, and could potentially spread SARS- CoV-2 to other patients and caregivers. This figure is similar to that reported by Kane et al. [12]. Detecting COVID-19 infected patients is a challenge; RT-PCR may be prone to sampling error and asymptomatic patients may have a lower viral load than symptomatic COVID-19 patients [13]. The benefit of chest computed tomography associated with RT-PCR is limited [14]. Moreover, chest CT is not recommended for screening in asymptomatic patients [15]. Fourteen days of isolation may improve screening of elective surgery patients; however, outliers may need more than 14 days [16].

Patients infected with SARS-CoV-2 have an increased risk of postoperative complications and mortality [1]. Five of the 25 Covid-19 infected patient (20 %) died. Covid-19 pneumonia was the cause of death in 3 patients from the COVID period. These figure are similar to those reported by other authors. Lei et al. [17] described postoperative outcomes in a group of 34 Covid-19 positive patients undergoing surgery - 44 % required admission to the ICU and 20.5 % died. An international cohort study reported a 30-day in-hospital mortality rate of 19 % in patients undergoing non-emergency surgery who were diagnosed with Covid-19 peri-operatively [2].

Due to protective protocols for both patients and health care personal and RT-PCR screening, no anaesthesiologists or surgical nurses were infected in the operating room during the pandemic period in the rugical theatre, but 3 surgeons had apositive RT-PCR in the same period.

There are some limitations to this study: first, because of its retrospective nature and the heterogeneity of the population, our results cannot be generalized to other hospitals. However, the data were collected automatically, double checked for postoperative complications, and are therefore reliable. Also, the severity and risk scores indicate that any bias introduced is more likely to occur in the COVID group.

## Conclusions

This study has showed the non-inferiority outcome of surgery performed in the COVID period; grade III to V complications and mortality slightly favours the COVID group. Our study shows that other hospitals can safely resume elective surgery during a pandemic if protective protocols for both patients and health care personal, RT-PCR screening, and 14 days quarantine for elective surgery patients are implemented. In summary, this study indicates that reassuming surgery in a pandemic is safe.

## Supplementary Information

**Additional file 1.**

## Data Availability

All study data is supplied in the tables attached to this article and in the supplementary material table. Please contact with the corresponding author (MC) if some data is requested.

## Abbreviations

SARS-CoV-2: severe acute respiratory syndrome coronavirus 2
COVID19: : coronavirus disease 2019
COVID: coronavirus disease
DRG: Diagnostic-related groups
WHO: World Health Organization
RT-PCR: REAL TIME-polymerase chain reaction
sICU: surgical intensive care unit
trauma-ICU: trauma-intensive care unit
OR: operating room
ICD-10AM: International Classification of Diseases and Related Health Problems, Tenth Revision, Australian Modification
