# Supplementary material for: Impact of the SARS-CoV-2 (COVID19) pandemic on the morbidity and mortality of high risk patients undergoing surgery: a non-inferiority retrospective observational study
Source: BMC Anesthesiol. 2021 Nov 26;21:295. doi: 10.1186/s12871-021-01495-3 (PMC8617361; doi:10.1186/s12871-021-01495-3)
Supplement: Supplementary file 1 — Additional file 1. [file 12871_2021_1495_MOESM1_ESM.docx]

Supplementary table 1: Mortality registered during PRECOVID and COVID (supplementary table)

| **Age (years)** | **Sex** | **Period** | **Diagnose** | **Procedure** | **Surgery**  **by Specialty** | **DRG s** | **DRG r** | **Length of stay (days)** | **Reintervention** | **Cause of death** | **COVID?** |
| --- | --- | --- | --- | --- | --- | --- | --- | --- | --- | --- | --- |
| 66.1 | 0 | 1 | Esophagic malignant tumor | Partial Esophagectomy | Digestive | 3 | 1 | 19.82 | No | Tracheoesophageal fistula | no |
| 56.4 | 1 | 1 | Toxicity due to corrosive substances | Exploratory laparotomy | Digestive | 3 | 2 | 0.53 | No | Intestinal ischemia | no |
| 66.0 | 0 | 1 | Cardiac assistance | Mitral valve replacement | Cardiovascular | 4 | 4 | 49.02 | Yes | Obstructive respiratory failure | no |
| 75.4 | 0 | 2 | Unspecified renal failure | Shoulder artrotomy | Orthopedics & Trauma | 4 | 4 | 34.58 | No | Respiratory failure | no |
| 85.0 | 1 | 1 | Infection/inflammation of internal prosthesis | Local tissue debridement of open fracture | Orthopedics & Trauma | 4 | 4 | 33.69 | No | Sepsis | no |
| 89.0 | 1 | 1 | Unspecified abdominal hernia | Exploratory laparotomy | Digestive | 3 | 3 | 5.64 | No | Incarcerated hernia | no |
| 69.4 | 0 | 2 | Unspecified spinal cord compression | Reduction of fracture | Orthopedics & Trauma | 4 | 3 | 31.25 | No | Respiratory failure | no |
| 86.5 | 0 | 1 | Non-traumatic subdural hemorrhage, chronic | Incision of cerebral meninges: drainage | Neurosurgery | 4 | 4 | 37.24 | No | Respiratory failure | no |
| 92.2 | 0 | 1 | Unspecified abdominal pain | Exploratory laparotomy | Digestive | 3 | 3 | 1.28 | No | Cardiac failure | no |
| 92.9 | 1 | 2 | Pathologic fracture of the right femur | Fracture reduction with internal fixation | Orthopedics & Trauma | 4 | 4 | 19.01 | No | Aspiration pneumonia | no |
| 76.6 | 0 | 1 | Cardiac assistance | Aortic valve replacement | Cardiovascular | 4 | 4 | 6.67 | No | Cerebral infarction | no |
| 63.6 | 0 | 2 | Bacteremia | Angioplasty or atherectomy of non-coronary vessels | Cardiovascular | 4 | 3 | 6.83 | No | Cardiorespiratory arrest | no |
| 78.9 | 0 | 2 | Non-traumatic subdural hemorrhage, chronic | Incision of cerebral meninges: drainage | Neurosurgery | 3 | 3 | 2.92 | No | Subdural hematoma | no |
| 66.3 | 0 | 1 | Unspecified malignant tumor | Supracondylar amputation | Cardiovascular | 4 | 4 | 18.64 | No | Respiratory Failure | no |
| 91.1 | 1 | 1 | Diabetic polyneuropathy | Toe amputation | Plastic & Neck | 4 | 4 | 46.27 | No | Cardiac failure | no |
| 82.8 | 0 | 2 | Unspecified abdominal pain | Exploratory laparotomy | Digestive | 3 | 3 | 27.18 | Yes | Respiratory failure | no |
| 78.3 | 0 | 1 | Larynx tumor | Partial laryngectomy | Plastic & Neck | 3 | 4 | 8.27 | Yes | Confusional disorder | no |
| 78.5 | 0 | 1 | Other disorders of the circulatory system | Exploratory laparotomy | Digestive | 3 | 3 | 0.15 | No | Intestinal ischemia | no |
| 64.2 | 1 | 2 | Interventricular septal defect | Extraction of cardiac assist device | Cardiovascular | 4 | 4 | 17.81 | No | Cardiogenic shock | no |
| 78.9 | 0 | 1 | Acute pancreatitis | Exploratory laparotomy | Digestive | 4 | 4 | 8.04 | No | sepsis | no |
| 53.6 | 0 | 1 | Lymphoma | Exploratory laparotomy | Digestive | 4 | 4 | 13.37 | No | Intestinal perforation | no |
| 85.1 | 0 | 1 | Trochanteric fracture; left femur | Femur fracture reduction with internal fixation | Orthopedics & Trauma | 3 | 4 | 7.12 | no | Respiratory failure | no |
| 72.2 | 0 | 1 | Infection and inflammatory reaction in cardiac valve prosthesis | Aortic valve replacement | Cardiovascular | 4 | 4 | 3.14 | no | Mitroaortic endocarditis | no |
| 81.2 | 1 | 1 | Unspecified iron deficiency anemia | Mitral valve replacement | Cardiovascular | 4 | 4 | 42.33 | no | Cardiac failure | no |
| 80.5 | 0 | 1 | Malignant tumor | Hemimaxillectomy (with bone graft or prosthesis) | Plastic & Neck | 3 | 3 | 19.23 | yes | Respiratory failure | no |
| 77.6 | 0 | 2 | Cerebral infarction | Supracondylar amputation | Cardiovascular | 3 | 3 | 24.90 | no | Acute pulmonary edema | no |
| 52.2 | 0 | 2 | Arteriovenous malformation | Exploratory laparotomy | Digestive | 4 | 4 | 4.23 | no | Hemorrhagic stroke | no |
| 76.5 | 1 | 1 | Atherosclerotic coronary artery | Aortocoronary bypass of three coronary arteries | Cardiovascular | 4 | 4 | 7.44 | no | Acute pulmonary edema | no |
| 62.9 | 0 | 1 | Myocardial infarction with ST-segment elevation | Aortocoronary bypass of three coronary arteries | Cardiovascular | 4 | 4 | 53.00 | yes | Refractory ventricular fibrillation | no |
| 79.6 | 0 | 1 | Left toe pain | Endoarterectomy of lower extremity arteries | Cardiovascular | 4 | 4 | 8.25 | yes | sepsis | no |
| 74.0 | 0 | 2 | Intestinal diverticulitis | Exploratory laparotomy | Digestive | 4 | 4 | 24.94 | no | Respiratory failure | no |
| 73.8 | 0 | 2 | Abdominal aortic aneurysm, with rupture | Intravascular implantation of other abdominal aorta grafts | Cardiovascular | 3 | 4 | 0.80 | no | Hemorrhagic shock | no |
| 74.8 | 0 | 1 | Malignant glottis tumor | Radical lymph node dissection | Plastic & Neck | 4 | 4 | 6.61 | no | Hemorrhagic shock | no |
| 75.5 | 1 | 1 | Bowel obstruction | Exploratory laparotomy | Digestive | 4 | 4 | 43.20 | yes | Intestinal fistula + respiratory failure | No |
| 81.0 | 0 | 1 | Cellulitis | Angioplasty of non-coronary vessels | Cardiovascular | 4 | 4 | 30.91 | No | Respiratory failure | No |
| 70.1 | 1 | 1 | Rheumatic aortic stenosis | Aortic valve replacement | Cardiovascular | 4 | 3 | 35.95 | No | Respiratory failure | no |
| 84.1 | 0 | 2 | Malignant tongue tumor | local excision or destruction of facial bone lesion | Plastic & Neck | 3 | 3 | 8.33 | No | Cardiorespiratory arrest | no |
| 33.1 | 1 | 1 | Unclassified hemorrhage | Arteriovenous fistula repair | Neurosurgery | 4 | 4 | 33.89 | Yes | Cerebral hematoma | no |
| 25.1 | 0 | 1 | Malignant cerebellar tumor | Other craniotomy | Neurosurgery | 4 | 4 | 92.84 | Yes | Respiratory failure | no |
| 77.2 | 1 | 2 | Infectious endocarditis | Aortic valve replacement | Cardiovascular | 3 | 4 | 1.87 | No | Cardiorespiratory arrest | no |
| 35.9 | 0 | 1 | Cardiac tamponade | Implantation of biventricular cardiac assist device | Cardiovascular | 4 | 3 | 3.42 | No | Cardiogenic shock | no |
| 45.3 | 0 | 2 | Unspecified pain | Exploratory laparotomy | Digestive | 4 | 4 | 27.52 | Yes | Sepsis | no |
| 77.8 | 0 | 1 | Unspecified abdominal pain | Exploratory laparotomy | Digestive | 4 | 4 | 162.96 | No | Respiratory failure | no |
| 72.2 | 0 | 1 | Malformation of the aqueduct of Sylvius | Other craniotomy | Neurosurgery | 4 | 4 | 48.57 | No | Ischemia and bleeding of vascular malformation | si |
| 70.8 | 0 | 2 | Acute pancreatitis | Other excision or destruction of pancreatic lesion | Digestive | 2 | 1 | 70.30 | No | Multiorgan failure | si |
| 85.8 | 1 | 1 | Periprosthetic fracture | Open fracture reduction with internal fixation | Orthopedics & Trauma | 4 | 4 | 51.79 | No | COVID respiratory failure | si |
| 79.3 | 0 | 2 | Abdominal aortic aneurysm, with rupture | Intravascular implantation of other abdominal aorta grafts | Cardiovascular | 4 | 4 | 15.43 | No | COVID respiratory failure | si |
| 73.6 | 0 | 2 | Incisional hernia, with obstruction | Other abdominal surgery | Digestive | 4 | 4 | 17.41 | No | COVID respiratory failure | si |

Sex (0: male, 1:female), Period (1: PRECOVID elective, 2: PRECOVID emergent, 3: COVID elective; 4: COVID emergent), DRGs: Diagnosis Related Groups severity, DRGr: Diagnosis Related Groups risk, COVID?: RT-PCR positive.
